# Effect of Polymer Degradation on Polymer Flooding in Heterogeneous Reservoirs

**DOI:** 10.3390/polym10080857

**Published:** 2018-08-02

**Authors:** Xiankang Xin, Gaoming Yu, Zhangxin Chen, Keliu Wu, Xiaohu Dong, Zhouyuan Zhu

**Affiliations:** 1 College of Petroleum Engineering, China University of Petroleum, Beijing 102249, China; xiankang.xin@hotmail.com (X.X.); zhachen@ucalgary.ca (Z.C.); dongxh@cup.edu.cn (X.D.); zhuzy02@cup.edu.cn (Z.Z.); 2 College of Petroleum Engineering, Yangtze University, Wuhan 430100, China; 3 Department of Chemical and Petroleum Engineering, University of Calgary, Calgary, AB T2N 1N4, Canada; wukeliu19850109@163.com

**Keywords:** polymer flooding, degradation, heterogeneous reservoir, numerical simulation

## Abstract

Polymer degradation is critical for polymer flooding because it can significantly influence the viscosity of a polymer solution, which is a dominant property for polymer enhanced oil recovery (EOR). In this work, physical experiments and numerical simulations were both used to study partially hydrolyzed polyacrylamide (HPAM) degradation and its effect on polymer flooding in heterogeneous reservoirs. First, physical experiments were conducted to determine basic physicochemical properties of the polymer, including viscosity and degradation. Notably, a novel polymer dynamic degradation experiment was recommended in the evaluation process. Then, a new mathematical model was proposed and an in-house three-dimensional (3D) two-phase polymer flooding simulator was designed to examine both polymer static and dynamic degradation. The designed simulator was validated by comparison with the simulation results obtained from commercial software and the results from the polymer flooding experiments. This simulator further investigated and validated polymer degradation and its effect. The results of the physical experiments showed that the viscosity of a polymer solution increases with an increase in polymer concentration, demonstrating their underlying power law relationship. Moreover, the viscosity of a polymer solution with the same polymer concentration decreases with an increase in the shear rate, demonstrating shear thinning. Furthermore, the viscosity of a polymer solution decreased with an increase in time due to polymer degradation, exhibiting an exponential relationship. The first-order dynamic degradation rate constant of 0.0022 day^−1^ was greater than the first-order static degradation rate constant of 0.0017 day^−1^. According to the simulation results for the designed simulator, a 7.7% decrease in oil recovery, after a cumulative injection volume of 1.67 pore volume (PV) was observed between the first-order dynamic degradation rate constants of 0 and 0.1 day^−1^, which indicates that polymer degradation has a detrimental effect on polymer flooding efficiency.

## 1. Introduction

Polymers are essential and ubiquitous in our daily life due to their broad range of properties [1]. They have been widely applied in the chemical, construction, agriculture, transportation, communications, aerospace, and medicine fields [2,3,4,5]. Polymers have played a particularly significant role, especially in the chemical industry [6]. Polymer flooding enhances oil recovery by reducing the water-oil mobility ratio [7], and this advanced technology has been used in oilfields since the 1950s [8]. The successful application of polymer flooding in the development of Chinese oilfields has alleviated the pressure to find more domestic oil and has helped meet the crude oil demand to a certain extent [9]. Therefore, polymer flooding is increasingly attracting attention, and its range of application is also growing [10].

Many researchers have conducted studies on the physicochemical properties of polymer to increase the efficiency of polymer flooding. These researchers have focused on the following five properties: (1) Viscosity. The viscosity of a polymer solution is determined by many factors, such as polymer concentration, polymer degradation, temperature, and the salinity of the water used in solution preparation [11,12]. (2) Rheological properties. Unlike a Newtonian fluid, a polymer solution often exhibits shear thinning performance within a certain range of shear rates [13,14]. (3) Inaccessible pore volumes (IPVs). Polymer is a molecular group with a certain hydraulic radius that cannot enter certain small pores during polymer flooding, resulting in an IPV [15], which affects the polymer flow in porous media. Polymer flow is influenced by permeability and polymer molecular weights [16,17]. (4) Absorption. In the process of polymer flooding, polymer inevitably undergoes adsorption in reservoirs due to its properties, surface properties of rock, and mineral composition, with adsorption rates that are affected by temperature and concentration [18]. (5) Degradation. Polymer degradation includes thermal, chemical, mechanical, and biological degradation [19,20], and its magnitude depends on temperature, oxygen content, a shear rate, and other factors [21,22].

Most of the above studies were well performed, but the research on polymer degradation is limited. This is a pressing need that must be addressed because polymer degradation can cause a polymer solution to decrease in viscosity, resulting in an increase in the water-oil mobility ratio [23]. This ratio is one of the key factors to successful polymer flooding. Many researchers investigated polymer degradation under static conditions or conditions different from reservoir conditions [24,25]. These experimental results cannot accurately describe polymer degradation during polymer flooding, especially when a polymer solution flows in reservoirs. This limitation is unlikely to yield correct results. Therefore, more detailed and accurate polymer degradation experiments must be designed.

Polymer flooding simulation was chosen due to its effectiveness in forecasting, estimating, and analyzing the factors that influence polymer flooding [26]. Most polymer physicochemical properties can be characterized [27]. However, despite the continuous improvement of numerical simulation research, one key problem has not yet been completely solved: how to reasonably and accurately describe the polymer degradation with a simulation. Polymer degradation was even directly ignored in previous polymer flooding models [28,29]. Moreover, commercial simulation software, such as ECLIPSE and the Computer Modeling Group (CMG) GEM, have also poorly treated polymer degradation [30]. To improve polymer flooding simulation, some methods have been proposed [31,32]. The first-order concentration attenuation model was used to characterize polymer concentration reduction during polymer flooding and could be embedded into the polymer mass conservation equations in simulation [33]. However, the model does not appropriately describe the process of polymer degradation in which the concentration remains the same and viscosity decreases, which is inconsistent with polymer theory. A viscosity reduction model could describe the variation in the polymer viscosity with time under static conditions [32,34], but the model was difficult to apply in polymer flooding simulations because it was unable to accurately obtain the polymer degradation time in each grid when a polymer solution flows. Although the concept of “time flux” was proposed to calculate an equivalent polymer degradation time [35], this method has no practical physical meaning, and cannot be validated by experiments. Thus, the calculation accuracy cannot be verified. Moreover, polymer static and dynamic degradations were not distinguished during simulation processes in these proposed methods. As a result, an accurate simulation result for polymer flooding is unlikely. Thus, designing a simulator that can reasonably and accurately describe polymer degradation with identifying the polymer static and dynamic degradations is necessary.

In this paper, physical experiments were performed to investigate the fundamental physicochemical properties of polymers, including viscosity and degradation performance. The viscosities of polymer solutions with different polymer concentrations were measured to estimate the increasing capacity of polymer viscosities. Moreover, the viscosity of a polymer solution with the same polymer concentration was measured at different shear rates. According to the two states of a polymer solution in a reservoir, non-flowing and flowing polymer solutions, the corresponding polymer static and dynamic experiments were designed. Notably, novel polymer dynamic degradation experiments were able to reproduce the condition of a polymer solution flowing in a reservoir, so the results were more accurate. In addition to the physical experiments, a new numerical method is proposed, and an in-house 3D two-phase polymer flooding simulator was designed to consider polymer degradation, where polymer static and dynamic degradations can be specifically identified. The validation of the designed simulator was conducted by comparing the simulation results run in the ECLIPSE V2013.1 software (Houston, TX, USA) and the results from the polymer flooding experiments. While considering polymer degradation, simulations were undertaken by applying the designed simulator to investigate the effect of polymer degradation on production indicators, including a pressure difference, water cut, and oil recovery. These results can assist investigations of polymer degradation effects on polymer flooding in heterogeneous reservoirs.

## 2. Methodology

### 2.1. Physical Experiments

#### 2.1.1. Materials

The properties of the materials including the oil sample, brine, polymer, and core samples are outlined in Table 1, Table 2, Table 3 and Table 4, respectively. Here, the single carbon number and the viscosity measurement of the oil sample were performed on an Agilent 7890A gas chromatograph from Agilent Technologies (Santa Clara, CA, USA) and the Physica MCR301 advanced rotary rheometer from Anton Paar (North Ryde, NSW, Australia).

#### 2.1.2. Polymer Solution Preparation and Composition

The temperature for polymer solution preparation was 25 °C, and its procedure was:

1. 199 mL of brine was placed in a 500 mL beaker, whose ion component concentrations can be seen in Table 2. The solution was stirred under 200 revolutions per minute (rpm) using a JJ-1B stirrer from Xinrui Instrument Factory (Changzhou, China).
2. 1.096 g of polymer was evenly added to the brine vortex for 30 s, whose properties are shown in Table 3.
3. The stirrer speed was reduced to 100 rpm and kept for 2 h.
4. The stirrer was stopped, and the polymer solution was deoxidized, sealed and statically stored in a brown glass bottle for 12 h.
5. Steps 1–4 was repeated, 400 mL of polymer solution with a concentration of 5000 mg/L was obtained.
6. 50 mL polymer solutions with a concentration of 5000 mg/L were diluted with 450, 200, 75, and 50 mL of brine, then 500, 250, 125, and 100 mL polymer solutions with concentrations of 500, 1000, 2000, and 2500 mg/L were obtained; 200 mL of polymer solution with a concentration of 5000 mg/L was diluted with 466.67 mL brine, then 666.67 mL of polymer solution with a concentration of 1500 mg/L was obtained.
7. After dilution, all polymer solutions were stirred at 100 rpm for 0.5 h using the JJ-1B stirrer.
8. The stirrer was stopped, and all polymer solutions were sheared under 16,900 rpm for 35 s using a Waring 7012S blender (Waring Products, Torrington, CT, USA) to simulate the degradation caused by a high shear rate in the near-wellbore region, which is called pre-shearing.
9. The blender was stopped, and all polymer solutions were deoxidized, sealed, and statically stored in brown glass bottles for 12 h.

#### 2.1.3. Viscometry

After preparing the polymer solution, the viscometric experiments were conducted on a Physica MCR301 advanced rotary rheometer from Anton Paar. The viscosity of the polymer solution with different concentrations was measured at a shear rate of 6.7 s^−1^, and the viscosity of the polymer solution with a concentration of 1500 mg/L was measured at different shear rates at 45 °C.

#### 2.1.4. Polymer Degradation Experiments

The polymer degradation experiments included polymer static and dynamic degradation experiments. The procedure for a polymer static degradation experiment was as follows:

1. After deoxidization, the prepared polymer solution with a concentration of 1500 mg/L was sealed in a stainless-steel tank like that used in the following polymer dynamic degradation experiment, and statically placed in a thermotank at a temperature of 45 °C.
2. 20 mL of the polymer solution was sampled after 1, 5, 15, 20, 40, 60, 80, 100, and 120 days, and its viscosity was measured at 45 °C. Notably, that oxygen was prevented from entering during the sampling process.

**Figure 1:**
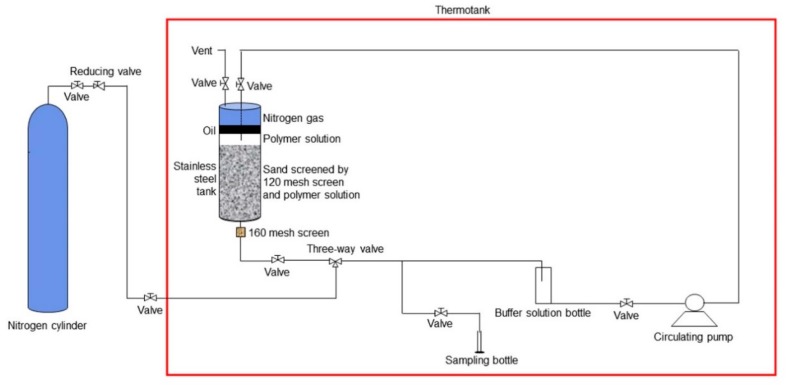
The schematic of a polymer dynamic degradation experiment.

The polymer dynamic degradation experiment was more complicated than a polymer static degradation experiment due to flowing. A schematic of the polymer dynamic degradation experiment is shown in Figure 1, and its experimental procedure was:

1. Sands were screened by a 120-mesh screen soaked with the prepared polymer solution for 2 days to complete polymer adsorption in a thermotank at a temperature of 45 °C to avoid the effect of polymer adsorption in the experiment.
2. Experimental devices were connected according to the schematic, and the temperature of the thermotank was maintained at 45 °C.
3. Deoxygenation of the entire system was conducted by filling the system with nitrogen gas to avoid the effect of oxygen.
4. A circulating pump was used to create the polymer solution flow through the sand layer at a flow rate of 1 m/day. Then 20 mL of the polymer solution was removed as a sample on day 1, 5, 15, 20, 40, 60, 80, 100, and 120, and its viscosity was measured at 45 °C. Avoiding oxygen in the sampling process also required careful handling.

#### 2.1.5. Polymer Flooding Experiments

**Figure 2:**
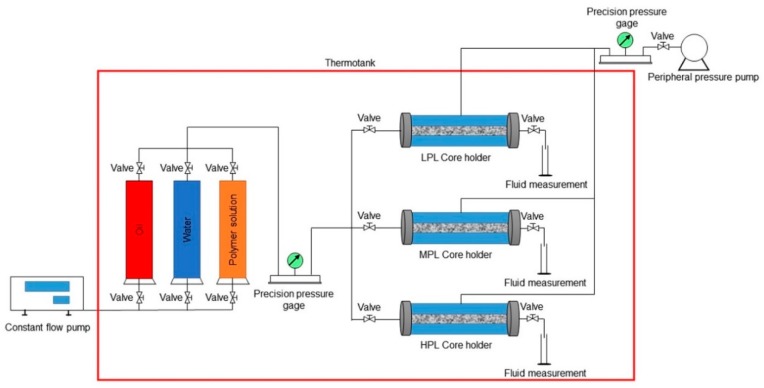
The schematic of a polymer flooding experiment.

The schematic of a polymer flooding experiment is shown in Figure 2. The experimental procedure follows:

1. Experimental devices were connected according to the schematic.
2. The temperature of the thermotank was set to 45 °C, and the cores were saturated with water for 24 h.
3. The water in the cores was displaced by the oil sample at a flow rate of 0.05 mL/min. The displacing flow rate was increased to 0.5 mL/min when the water cut at the outlet was lower than 2%, until the volume of the injected oil sample reached 10 times the PV of the cores, and no water was produced. Then, the constant flow pump was stopped, and this condition was maintained for 24 h.
4. The polymer solution was used to displace at a constant flow rate of 0.64 mL/min until the volume of the injected polymer solution reached 0.64 PV. Then, the constant flow pump was stopped after the polymer flooding, and this condition was maintained for 120 days.
5. Water was sequentially used to displace at a constant flow rate of 0.64 mL/min until the volume of the injected water reached 4.16 PV. After the subsequent water flooding, the constant flow pump was stopped.

To ensure that the experiment was not affected by air, especially oxygen, the relevant experimental devices including the pipelines were completely filled with the corresponding liquid during the experiment.

### 2.2. Mathematical Model

#### 2.2.1. Assumptions

The assumptions for the mathematical model include:

1. Only oil and water phases were present, and there was no mass exchange between them.
2. The flow process was isothermal and the flow followed Darcy’s law.
3. The fluids were compressible, and the rock was compressible and anisotropic.
4. Polymer components were only divided into high and low molecular weight polymer components. The viscosity of the polymer solution was determined by the high molecular weight polymer component, and the virgin polymer solution only had the high molecular weight polymer component.
5. The mixture of water and polymer was ideal, and they existed only in the water phase.
6. The effects of capillary force and gravity were considered.

#### 2.2.2. Treatment of Mechanisms

In the polymer injection process, changes will appear. These include changes in the viscosity of the water phase, polymer adsorption, the permeability reduction of the water phase, an inaccessible pore volume, and polymer degradation. For the treatment of the viscosity of the water phase, an effective polymer viscosity was introduced by Todd and Longstaff [36]. According to their method, the mixing parameter was set to one, which meant that the polymer and water were ideally mixed, and the effective polymer viscosity and the effective water viscosity were equal. The polymer adsorption is considered a Langmuir adsorption isotherm [37,38]:

(1) $$c_{ap}=c_{apmax}\frac{b_{p}c_{p}}{1+b_{p}c_{p}}$$

where $c_{ap}$ is the adsorbed concentration of polymer in kg/kg, $c_{apmax}$ is the maximum adsorbed concentration of polymer in kg/kg, $b_{p}$ is the adsorption coefficient, and $c_{p}$ is the polymer concentrations in kg/m^3^. The permeability reduction factor of the water phase $R_{k}$ follows [39]:

(2) $$R_{k}=1+\left(\mathrm{RRF}-1\right)\frac{c_{ap}}{c_{apmax}}$$

where RRF is the residual resistance factor, defined as the ratio between the water permeability measured before and after the polymer flooding. The inaccessible pore volume factor $f_{ipv}$ was modeled using a constant. The computational expression of the first-order degradation rate constant $R_{pd}$ is:

(3) $$R_{pd}=-\frac{dc_{hp}/c_{hp}}{dt_{pd}}$$

where $R_{pd}$ is in day^−1^, d is the derivative symbol, $c_{hp}$ is the high molecular weight polymer concentration in kg/m^3^, and $t_{pd}$ is the polymer degradation time in day. Here, it was also modeled using a constant.

#### 2.2.3. Mass Conservation Equations

According to Darcy’s law and the conservation of mass, the mass conservation equations of all components can be obtained as follows:

For oil:

(4) $$\nabla \cdot \left[\frac{\vec{k}k_{ro}}{\mu_{o}B_{o}}\left(\nabla p_{o}-\rho_{o}g\nabla D\right)\right]+q_{o}=\frac{\partial }{\partial t}\left(\frac{\phi s_{o}}{B_{o}}\right)$$

For water:

(5) $$\nabla \cdot \left[\frac{\vec{k}k_{rw}}{\mu_{we}B_{w}R_{k}}\left(\nabla p_{w}-\rho_{w}g\nabla D\right)\right]+q_{w}=\frac{\partial }{\partial t}\left(\frac{\phi s_{w}}{B_{w}}\right)$$

For the high molecular weight polymer component:

(6) $$\begin{array}{l} \nabla \cdot \left[\frac{\vec{k}k_{rw}c_{hp}}{\mu_{we}B_{w}R_{k}}\left(\nabla p_{w}-\rho_{w}g\nabla D\right)\right]-c_{hp}R_{pd}+q_{w}c_{hp}\\ \;=\frac{\partial }{\partial t}\left[\frac{\phi \left(1-f_{ipv}\right)s_{w}c_{hp}}{B_{w}}\right]+\frac{\partial \left[\left(1-f_{ipv}\right)\left(1-\phi \right)\rho_{r}c_{hpa}\right]}{\partial t} \end{array}$$

For the low molecular weight polymer component:

(7) $$\begin{array}{l} \nabla \cdot \left[\frac{\vec{k}k_{rw}c_{lp}}{\mu_{we}B_{w}R_{k}}\left(\nabla p_{w}-\rho_{w}g\nabla D\right)\right]+c_{hp}R_{pd}+q_{w}c_{lp}\\ \;=\frac{\partial }{\partial t}\left[\frac{\phi \left(1-f_{ipv}\right)s_{w}c_{lp}}{B_{w}}\right]+\frac{\partial \left[\left(1-f_{ipv}\right)\left(1-\phi \right)\rho_{r}c_{lpa}\right]}{\partial t} \end{array}$$

where ∇ is the divergence operator, $\vec{k}$ is the absolute permeability tensor in μm^2^, $k_{ro}$ and $k_{rw}$ are the relative permeabilities of oil and water, respectively; $\mu_{o}$ is the viscosity of the oil phase in mPa·s; $\mu_{we}$ is the effective viscosity of the water phase in mPa·s; $B_{o}$ and $B_{w}$ are the oil and water formation volume factors in m^3^/m^3^, respectively; $p_{o}$ and $p_{w}$ are the pressures of the oil and water phases in Pa, respectively; $\rho_{o}$ and $\rho_{w}$ are the oil and water densities in kg/m^3^, respectively; and g is the gravitational acceleration in m/s^2^. D is the vertical height in m and $q_{o}$ and $q_{w}$ are the source/sink terms for the oil and water phases in m^3^/(day·m^3^) respectively. The source term is negative, and the sink term is positive. ∂ is the symbol used to denote partial derivatives, and t is time in days. ϕ is the porosity; $s_{o}$ and $s_{w}$ are the oil and water phase saturation, respectively; $c_{lp}$ is the low molecular weight polymer concentration in kg/m^3^; $f_{ipv}$ is the inaccessible pore volume factor; $\rho_{r}$ is the rock density in kg/m^3^; and $c_{hpa}$ and $c_{lpa}$ are the adsorption concentrations of the high and low molecular weight polymers in kg/kg, respectively.

From the mass conservation Equations (6) and (7), the high molecular weight polymer component degraded into the low molecular weight polymer, but the total polymer concentration was retained and followed the conservation of mass, which demonstrated that the theory of polymer chemistry was followed in the model establishment process.

#### 2.2.4. Auxiliary Equations and Equations of State

The auxiliary equations and equations of state are as follows. The auxiliary equations include:

(8) $$s_{o}+s_{w}=1$$

(9) $$p_{cow}\left(s_{w}\right)=p_{o}-p_{w}$$

where $p_{cow}\left(s_{w}\right)$ is the capillary pressure in the water-oil system in Pa, which is a function of the water phase saturation. The equations of state include:

(10) $$k_{ro}=k_{ro}\left(s_{w}\right)$$

(11) $$k_{rw}=k_{rw}\left(s_{w}\right)$$

(12) $$\rho_{o}=\rho_{o}\left(p_{o}\right)$$

(13) $$\rho_{w}=\rho_{w}\left(p_{w}\right)$$

(14) $$\phi =\phi \left(p_{r}\right)$$

(15) $$\mu_{we}=\mu_{we}\left(c_{hp},v_{p}\right)$$

(16) $$R_{pd}=R_{pd}\left(v_{p}\right)$$

where $p_{r}$ is the reservoir pressure in Pa, and $v_{p}$ is the velocity of a polymer solution in m/day. Equation (15) shows that the effective viscosity of the water phase is not only a function of the high molecular weight polymer concentration but also a function of the polymer solution velocity. Thus, the relationship between the viscosity of the polymer solution and shear rate, one of the rheological properties of the polymer solution, can be characterized in a mathematical model. In addition, the effective viscosity of the water phase was set to be equal to the viscosity of the brine/water when the high molecular weight polymer concentration was zero, which is not only more realistic but also avoids the effective viscosity of the water phase of zero in the calculation process. Equation (16) shows the first-order degradation rate constant as a function of the polymer solution velocity. According to whether the polymer solution flow rate is zero, the first-order degradation rate constant can be divided into first-order static and dynamic degradation rate constants. Therefore, the polymer static and dynamic degradation can be simultaneously described in the mathematical model.

#### 2.2.5. Initial and Boundary Conditions

The initial conditions include the distribution of initial pressure, saturation and high and low molecular weight polymer concentrations:

(17) $$p_{r}\left(x,y,z\right){|}_{t=0}=p_{ri}\left(x,y,z\right)$$

(18) $$s_{w}\left(x,y,z\right){|}_{t=0}=s_{wi}\left(x,y,z\right)$$

(19) $$c_{hp}\left(x,y,z\right){|}_{t=0}=c_{hpi}\left(x,y,z\right)$$

(20) $$c_{lp}\left(x,y,z\right){|}_{t=0}=c_{lpi}\left(x,y,z\right)$$

where (x,y,z) are the coordinates, $p_{ri}$ is the initial reservoir pressure in Pa, $s_{wi}$ is the initial water saturation, and $c_{hpi}$ and $c_{lpi}$ are the initial high and low molecular weight polymer concentrations in kg/m^3^, respectively. The outer boundary is a closed boundary with no-flow:

(21) $$\frac{\partial p}{\partial n}{|}_{B}=f\left(x,y,z,t\right)=0$$

where $\frac{\partial p}{\partial n}{|}_{B}$ denotes the derivative of the boundary pressure in the direction of the outer normal. The inner boundary conditions are as follows:

(22) $$Q_{l}\left(x,y,z,t\right){|}_{{\left(x,y,z\right)}_{well}}=Q_{l}\left(t\right)$$

(23) $$c_{i}\left(x,y,z,t\right){|}_{{\left(x,y,z\right)}_{well}}=c_{i}\left(t\right)$$

where $Q_{l}$ is the flow rate of phase l, which can be calculated by Peaceman’s well model [40]; ${\left(x,y,z\right)}_{well}$ is the grid coordinate of a well; $c_{i}$ is the concentration of the component i; l represents the oil or water phase, o represents the oil phase; w represents the water phase; i represents the high or low molecular weight polymer; hp represents the high molecular weight polymer; and lp represents the low molecular weight polymer. 

#### 2.2.6. Solution Method

Because the mathematical equations were complex, obtaining an analytical solution was difficult [41]. Here, the control volume finite difference method, which is a numerical method, was applied to obtain a solution, and a block-centered grid was employed as the grid system. The discretized forms of the mass conservation equations are as follows:

(24) $$\begin{array}{ll} {\left(T\lambda_{o}\Delta \Phi_{o}\right)}_{i+1/2,j,k}^{n+1} & -{\left(T\lambda_{o}\Delta \Phi_{o}\right)}_{i-1/2,j,k}^{n+1}+{\left(T\lambda_{o}\Delta \Phi_{o}\right)}_{i,j+1/2,k}^{n+1}-{\left(T\lambda_{o}\Delta \Phi_{o}\right)}_{i,j-1/2,k}^{n+1}\\ & +{\left(T\lambda_{o}\Delta \Phi_{o}\right)}_{i,j,k+1/2}^{n+1}-{\left(T\lambda_{o}\Delta \Phi_{o}\right)}_{i,j,k-1/2}^{n+1}+Q_{oi,j,k}^{n+1}\\ & =\left[{\left(\frac{v\phi s_{o}}{B_{o}}\right)}_{i,j,k}^{n+1}-{\left(\frac{v\phi s_{o}}{B_{o}}\right)}_{i,j,k}^{n}\right]/\Delta t \end{array}$$

(25) $$\begin{array}{cc} {\left(T\lambda_{w}\Delta \Phi_{w}\right)}_{i+1/2,j,k}^{n+1} & -{\left(T\lambda_{w}\Delta \Phi_{w}\right)}_{i-1/2,j,k}^{n+1}+{\left(T\lambda_{w}\Delta \Phi_{w}\right)}_{i,j+1/2,k}^{n+1}-{\left(T\lambda_{w}\Delta \Phi_{w}\right)}_{i,j-1/2,k}^{n+1}\\ + & {\left(T\lambda_{w}\Delta \Phi_{w}\right)}_{i,j,k+1/2}^{n+1}-{\left(T\lambda_{w}\Delta \Phi_{w}\right)}_{i,j,k-1/2}^{n+1}+Q_{wi,j,k}^{n+1}\\ = & \left[{\left(\frac{v\phi s_{w}}{B_{w}}\right)}_{i,j,k}^{n+1}-{\left(\frac{v\phi s_{w}}{B_{w}}\right)}_{i,j,k}^{n}\right]/\Delta t \end{array}$$

(26) $$\begin{array}{ll} {\left(T\lambda_{w}c_{hp}\Delta \Phi_{w}\right)}_{i+1/2,j,k}^{n+1} & -{\left(T\lambda_{w}c_{hp}\Delta \Phi_{w}\right)}_{i-1/2,j,k}^{n+1}+{\left(T\lambda_{w}c_{hp}\Delta \Phi_{w}\right)}_{i,j+1/2,k}^{n+1}\\ & \;-{\left(T\lambda_{w}c_{hp}\Delta \Phi_{w}\right)}_{i,j-1/2,k}^{n+1}+{\left(T\lambda_{w}c_{hp}\Delta \Phi_{w}\right)}_{i,j,k+1/2}^{n+1}-{\left(T\lambda_{w}c_{hp}\Delta \Phi_{w}\right)}_{i,j,k-1/2}^{n+1}\\ & \;-{\left(vc_{hp}R_{pd}\right)}_{i,j,k}^{n+1}+{\left(Q_{w}c_{hp}\right)}_{i,j,k}^{n+1}\\ & \;=\{{\left[\frac{v\phi \left(1-f_{ipv}\right)s_{w}c_{hp}}{B_{w}}\right]}_{i,j,k}^{n+1}-{\left[\frac{v\phi \left(1-f_{ipv}\right)s_{w}c_{hp}}{B_{w}}\right]}_{i,j,k}^{n}\\ & \;+{\left[v\left(1-f_{ipv}\right)\left(1-\phi \right)\rho_{r}c_{hpa}\right]}_{i,j,k}^{n+1}-{\left[v\left(1-f_{ipv}\right)\left(1-\phi \right)\rho_{r}c_{hpa}\right]}_{i,j,k}^{n}\}/\Delta t \end{array}$$

(27) $$\begin{array}{ll} {\left(T\lambda_{w}c_{lp}\Delta \Phi_{w}\right)}_{i+1/2,j,k}^{n+1} & -{\left(T\lambda_{w}c_{lp}\Delta \Phi_{w}\right)}_{i-1/2,j,k}^{n+1}+{\left(T\lambda_{w}c_{lp}\Delta \Phi_{w}\right)}_{i,j+1/2,k}^{n+1}\\ & \;-{\left(T\lambda_{w}c_{lp}\Delta \Phi_{w}\right)}_{i,j-1/2,k}^{n+1}+{\left(T\lambda_{w}c_{lp}\Delta \Phi_{w}\right)}_{i,j,k+1/2}^{n+1}-{\left(T\lambda_{w}c_{lp}\Delta \Phi_{w}\right)}_{i,j,k-1/2}^{n+1}\\ & \;+{\left(vc_{hp}R_{pd}\right)}_{i,j,k}^{n+1}+{\left(Q_{w}c_{lp}\right)}_{i,j,k}^{n+1}\\ & \;=\{{\left[\frac{v\phi \left(1-f_{ipv}\right)s_{w}c_{lp}}{B_{w}}\right]}_{i,j,k}^{n+1}-{\left[\frac{v\phi \left(1-f_{ipv}\right)s_{w}c_{lp}}{B_{w}}\right]}_{i,j,k}^{n}\\ & \;+{\left[v\left(1-f_{ipv}\right)\left(1-\phi \right)\rho_{r}c_{lpa}\right]}_{i,j,k}^{n+1}-{\left[v\left(1-f_{ipv}\right)\left(1-\phi \right)\rho_{r}c_{lpa}\right]}_{i,j,k}^{n}\}/\Delta t \end{array}$$

where n is the time step number and (i,j,k) is the grid block number. $T_{i+1/2,j,k}=\frac{2{\left(dydzk_{xx}\right)}_{i,j,k}{\left(dydzk_{xx}\right)}_{i+1,j,k}}{{\left(dydzk_{xx}\right)}_{i,j,k}dx_{i+1,j,k}+{\left(dydzk_{xx}\right)}_{i+1,j,k}dx_{i,j,k}}$ is a conductivity coefficient in the x direction between the grid blocks (i,j,k) and (i+1,j,k) in um^2^·m; in which dx,dy, and dz are the length of the grid block in the x, y and z directions in m, respectively; and $k_{xx}$ is the absolute permeability in the x direction. $\Delta \Phi_{oi+1/2,j,k}=p_{oi+1,j,k}-p_{oi,j,k}+\frac{1}{2}\left(\rho_{oi+1,j,k}+\rho_{oi,j,k}\right)g\left(D_{i+1,j,k}-D_{i,j,k}\right)$ is the potential difference in Pa, $\lambda_{oi+1/2,j,k}=\{\begin{array}{c} {\left(\frac{k_{ro}}{\mu_{o}B_{o}}\right)}_{i+1,j,k}\;if\;\Delta \Phi_{oi+1/2,j,k}\geq 0\\ {\left(\frac{k_{ro}}{\mu_{o}B_{o}}\right)}_{i,j,k}\;if\;\Delta \Phi_{oi+1/2,j,k}<0 \end{array}$ and $\lambda_{wi+1/2,j,k}=\{\begin{array}{c} {\left(\frac{k_{rw}}{\mu_{we}B_{w}R_{k}}\right)}_{i+1,j,k}\;if\;\Delta \Phi_{wi+1/2,j,k}\geq 0\\ {\left(\frac{k_{rw}}{\mu_{we}B_{w}R_{k}}\right)}_{i+1,j,k}\;if\;\Delta \Phi_{wi+1/2,j,k}<0 \end{array}$ are the mobilities for the oil and water phases in the x direction between the grid blocks (i,j,k) and (i+1,j,k) in mPa·s^−1^. Tλ can be called transmissibility; $Q_{oi,j,k}$ and $Q_{wi,j,k}$ are the oil and water flow rates in the grid block (i,j,k) under the ground standard conditions in m^3^/day, respectively. Production is negative, and injection is positive. $v_{i,j,k}$ is the volume of the grid block (i,j,k). Similar quantities can be obtained but are not presented here.

**Figure 3:**
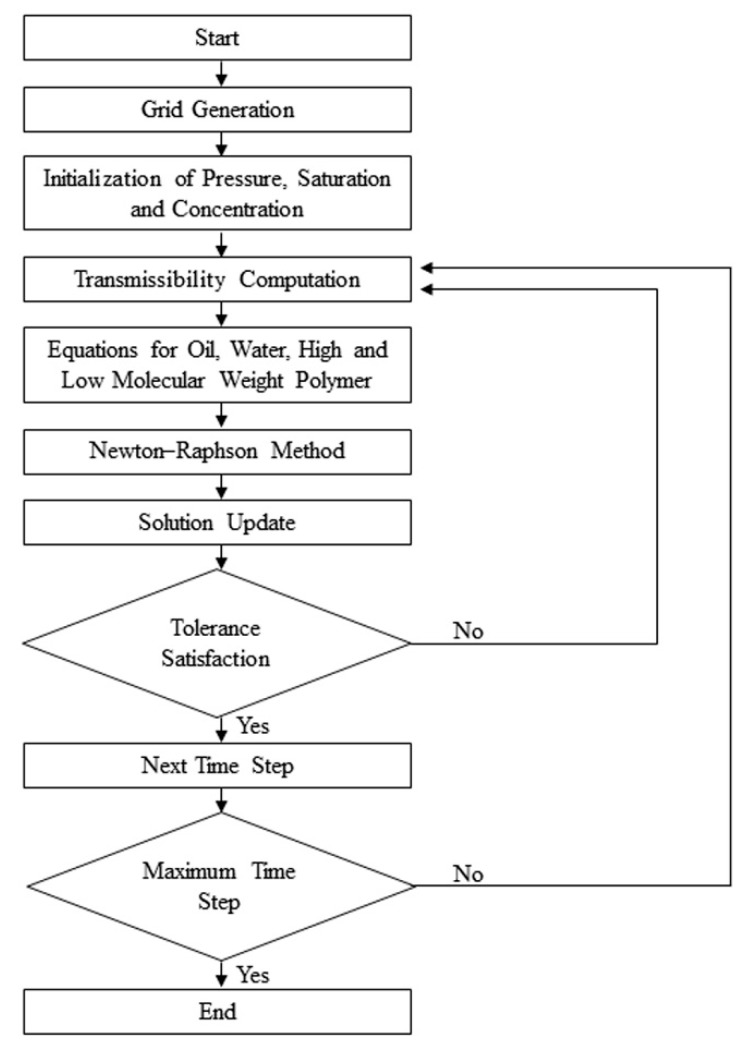
The solution flow chart.

Equations (24)–(27) constitute a system of nonlinear equations. To ensure the stability of computation, the full implicit method was applied to solve the nonlinear equations. For the solution of each specific time step, the Newton-Raphson method, an iterative method, was used. The solution flow chart is shown in Figure 3. More details can be found in Chen’s works [42,43]. Finally, pressure was obtained along with production, oil, and water phase saturation distribution, as well as the high and low molecular weight polymer concentrations.

## 3. Results and Discussion

### 3.1. Viscosity of Polymer Solution

**Figure 4:**
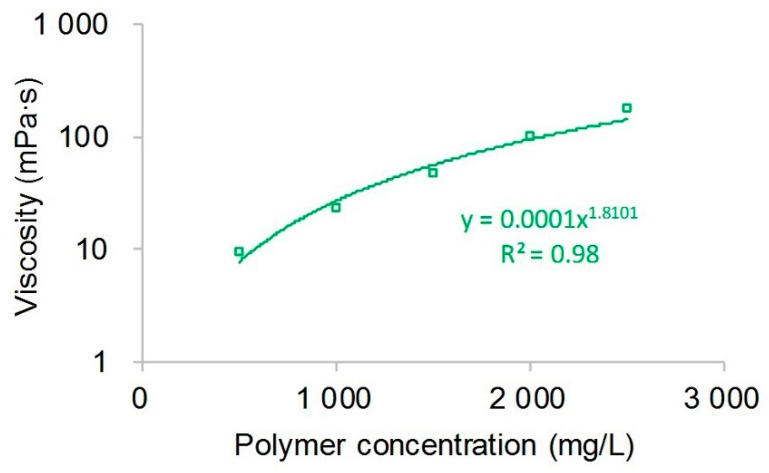
The relationship between the viscosity of the polymer solution and polymer concentration.

**Figure 5:**
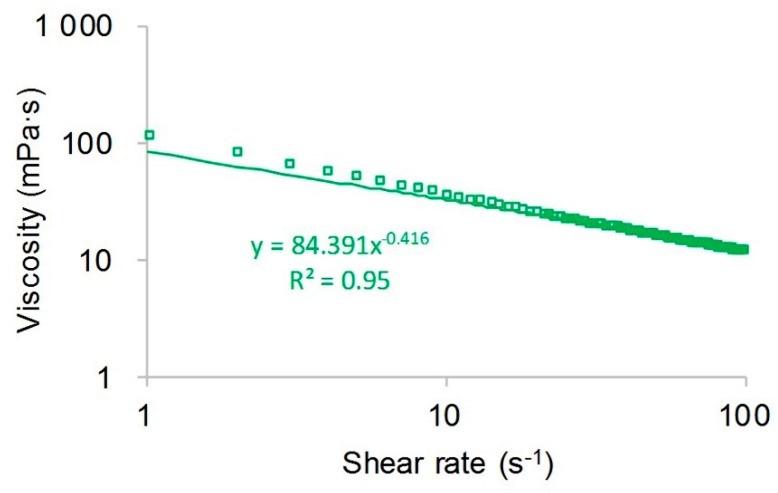
Shear thinning of polymer solution with a concentration of 1500 mg/L.

The relationship between the viscosity of the polymer solution and polymer concentration is presented in Figure 4. Clearly, relationship shows a good power law relationship, and the square of the correlation coefficient (*R*^2^) reached 0.98. Their relationship expression can be written as:

(28) $$\mu_{ps}=0.0001\times c_{p}^{1.8101}$$

where $\mu_{ps}$ is the viscosity of the polymer solution in mPa·s and $c_{p}$ is the polymer concentration in mg/L. From the plot, the polymer has an excellent viscosity-increasing performance, and the viscosity of the polymer solution significantly increased with an increase in polymer concentration. The viscosity of the polymer solution with a polymer concentration of 2500 mg/L was nearly 17 times greater than that of the polymer solution with a polymer concentration of 500 mg/L. The main reason for this finding is that the polymer solution with a higher polymer concentration has longer molecular chains and more entanglements, resulting in a larger hydrodynamic radius, which increases the viscosity of the polymer solution [44,45,46]. However, the viscosity of the polymer solution with a concentration of 1500 mg/L decreased with an increase in shear rate, which can be seen in Figure 5. The viscosity of the polymer solution at 100 s^−1^ was about one-fourth that of the polymer solution at 6.7 s^−1^. The main reason for the shear thinning of the polymer solution is that a higher shear rate caused the intertwined polymer molecules to collapse, resulting in a decrease in the hydrodynamic radius, which is followed by a reduction in the viscosity of the polymer solution [47,48].

### 3.2. Polymer Degradation

**Figure 6:**
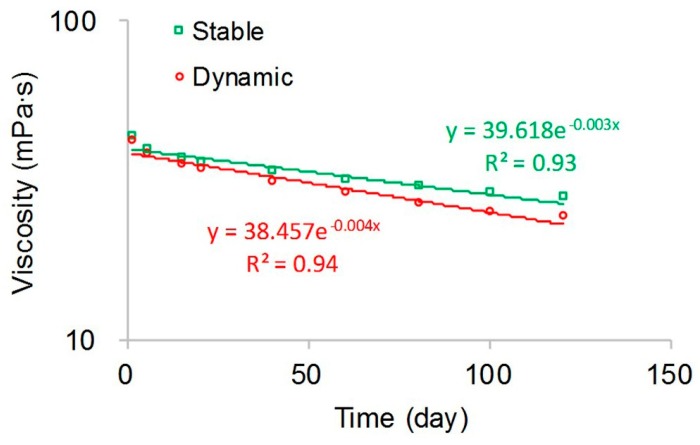
Polymer static and dynamic degradation curves.

Polymer static and dynamic degradations are illustrated in Figure 6. Unlike the relationship between the viscosity of the polymer solution and polymer concentration, the relationship of polymer static and dynamic degradations showed an exponential relationship with correlation coefficient squares of 0.93 and 0.94, which, for polymer static degradation, is:

(29) $$\mu_{ps}=39.618\times e^{-0.003t_{pd}}$$

and for polymer dynamic degradation is:

(30) $$\mu_{ps}=38.457\times e^{-0.004t_{pd}}$$

The viscosity of the polymer solution decreased with increasing time. The viscosity of the polymer at 120 days was about three-fifths or 60% of the virgin polymer solution during polymer static degradation, and the viscosity of the polymer at 120 days was approximately half that of the virgin polymer solution during polymer dynamic degradation. The main reason for this finding is that the long molecular polymer chains break down in the process of polymer degradation, and the length of average molecular chains is cut along with the average molecular weight of polymer, which leads to a decrease in the viscosity of the polymer solution [19,49]. Combining the polymer viscometric experiment and the polymer degradation experiment results, the first-order static and dynamic degradation rate constants can be calculated. Compared with the first-order static degradation rate constant of 0.0017 day^−1^, the first-order dynamic degradation rate constant was 0.0022 day^−1^, which was greater than the first-order static degradation rate constant. This occurred because the additional shear stress led to additional chain ruptures as the polymer solution flowed through the sands, resulting in a decrease in the viscosity of the polymer solution and an increase in the polymer degradation rate [50,51].

### 3.3. Numerical Simulation

#### 3.3.1. Validation

**Figure 7:**
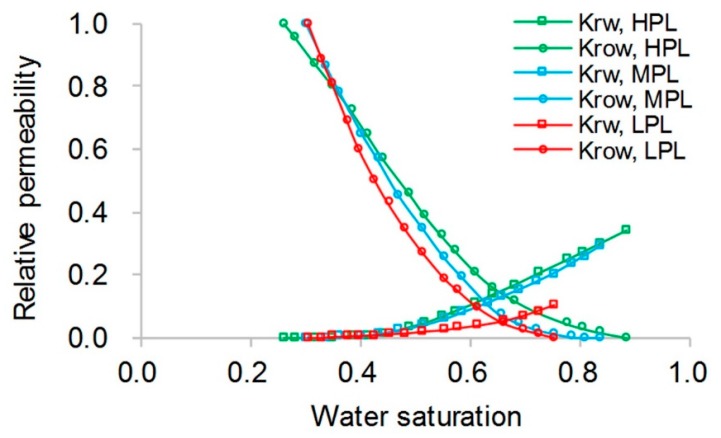
Relative permeabilities without polymer degradation.

**Figure 8:**
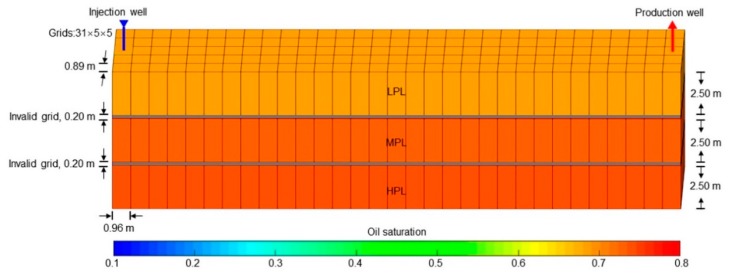
Grid system, well location, and 3D distributions of oil saturation in the initial state for the case without polymer degradation.

**Figure 9:**
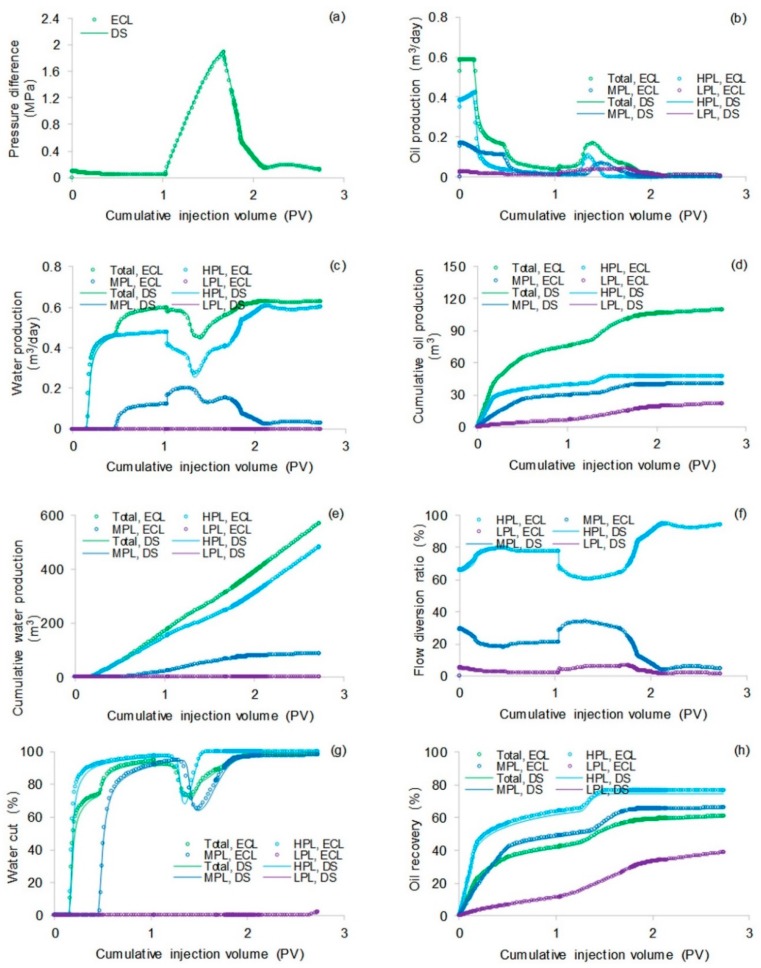
Comparisons of results in terms of: (**a**) pressure difference, (**b**) oil production, (**c**) water production, (**d**) cumulative oil production, (**e**) cumulative water production, (**f**) flow diversion ratio, (**g**) water cut, and (**h**) oil recovery using ECLIPSE V2013.1 software and designed simulator for the situation without polymer degradation.

**Figure 10:**
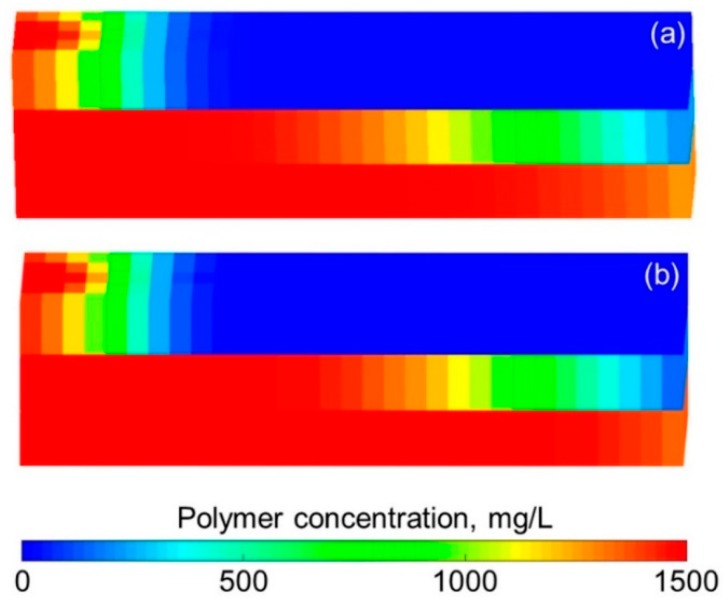
Comparison of 3D polymer concentration distributions after cumulative injection volume of 1.67 PV of (**a**) ECLIPSE V2013.1 software and (**b**) designed simulator in running the case without polymer degradation.

**Figure 11:**
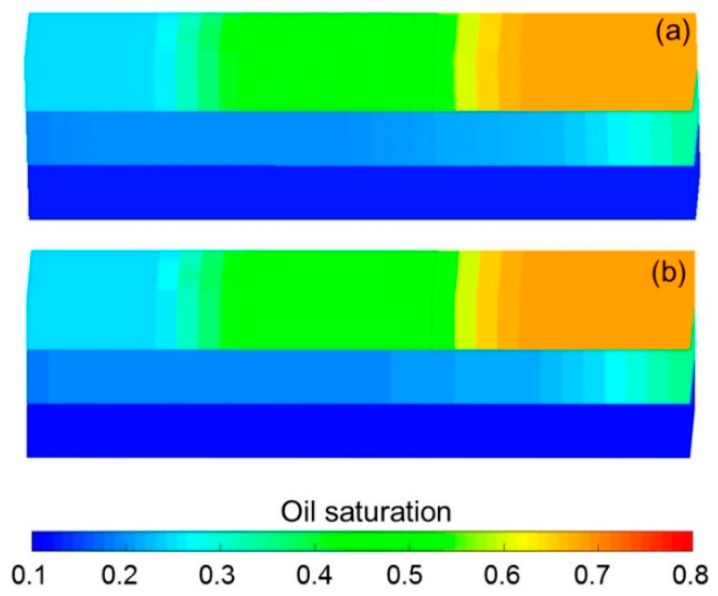
Comparison of 3D oil saturation distributions after cumulative injection volume of 1.67 PV obtained with (**a**) ECLIPSE V2013.1 software and (**b**) designed simulator in running the case without polymer degradation.

The simulation results obtained with the ECLIPSE V2013.1 (ECL) software were used as a comparison with our designed simulator (DS) to validate the method without polymer degradation because it is a recognized commercial numerical reservoir simulator, and its simulation results are authoritative [52]. The software’s reservoir properties, fluid properties, relative permeabilities, and initial conditions were the same as those in the polymer flooding experiment in this paper, as shown in Table 5. Moreover, the other main input parameters, including production data, grid parameters, and well location, were also the same and are provided in Table 5. Figure 7, Figure 8 and Figure 9 compare the results of the production indicators, including pressure differences, oil production, water production, water cut, cumulative oil production, cumulative water production, and oil recovery of the ECLIPSE V2013.1 software and the designed simulator. Clearly, the simulation results were very close, and the difference for each production indicator was less than 0.2%. Figure 10 and Figure 11 present the 3D polymer concentration distributions and remaining oil saturation distributions after a cumulative injection volume of 1.67 PV (i.e., 1.03 PV water injection during water flooding and 0.64 PV polymer solution injection during polymer flooding) monitored by the ECLIPSE V2013.1 software and the designed simulator, respectively. From the overall polymer concentration distribution and remaining oil saturation distribution data, the results of both the ECLIPSE V2013.1 software and our designed simulator were also similar. Thus, the validation of the designed simulator without polymer degradation was confirmed, demonstrating both high accuracy and reliability.

However, no widely accepted commercial software considers polymer degradation [33], and conducting simulations with consideration of both polymer static and dynamic degradation was not possible. Therefore, we compared the simulation results of our designed simulator with the data of an actual polymer flooding experiment for validation, where both polymer static and dynamic degradation were considered.

**Figure 12:**
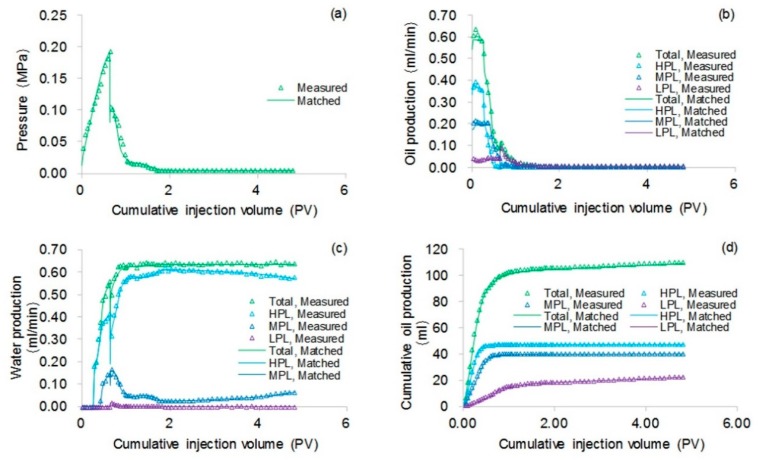

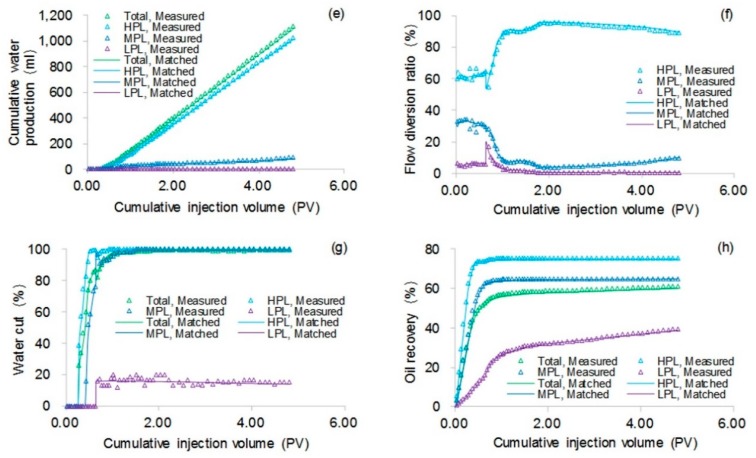
Comparison results in terms of: (**a**) pressure difference, (**b**) oil production, (**c**) water production, (**d**) cumulative oil production, (**e**) cumulative water production, (**f**) flow diversion ratio, (**g**) water cut, and (**h**) oil recovery of the polymer flooding experiment obtained by the designed simulator in simulating the polymer flooding experiment.

**Figure 13:**
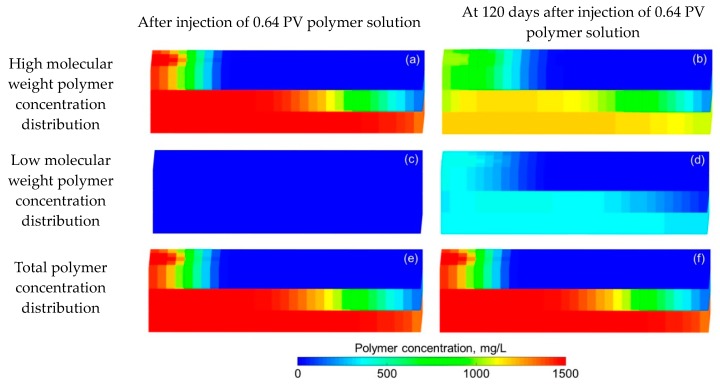
3D (**a**) high molecular weight, (**c**) low molecular weight, and (**e**) total polymer concentration distributions after injection of 0.64 PV polymer solution, and 3D (**b**) high molecular weight, (**d**) low molecular weight, and (**f**) total polymer concentration distributions at 120 days after injection of 0.64 PV polymer solution by the designed simulator in simulating the polymer flooding experiment.

**Figure 14:**
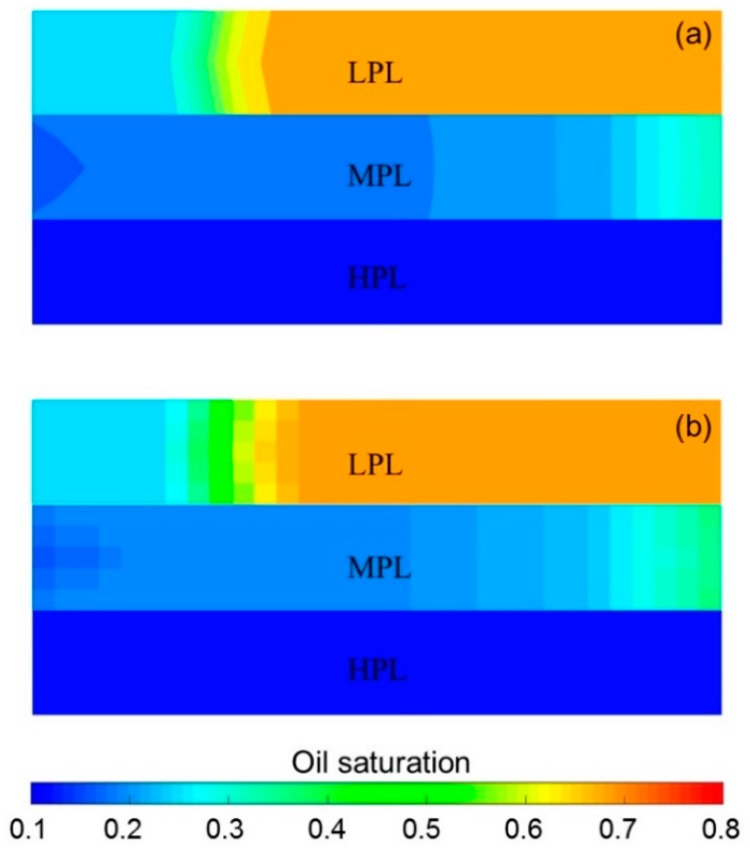
Oil saturation distributions of each layer after injection of 0.64 PV polymer solution obtained with (**a**) the saturation detector and (**b**) the designed simulator.

Some parameters, including the reservoir properties, fluid properties, relative permeabilities, and initial conditions, were the same as those in the case for validation without polymer degradation, not repeatedly presented here. The different parameters for the experimental simulation from the validation case without polymer degradation are provided in Table 6. The comparative results of the pressure difference, oil production, water production, water cut, cumulative oil production, and oil recovery are indicated in Figure 12. This figure reveals that the simulation result was close to the experimental result. There were differences of 1.00% in water cut and 1.70% in oil recovery after a cumulative injection volume of 1.67 PV, and differences of only 0.08% in water cut and 2.07% in oil recovery were recorded when a 4.8 PV cumulative injection volume was used. Figure 13 illustrates the 3D high molecular weight, low molecular weight, and total polymer concentration distributions after injection of 0.64 PV polymer solution, and at 120 days after injection of 0.64 PV polymer solution by the designed simulator. Figure 13 shows that the high molecular weight polymer concentration decreased, which means that the high molecular weight polymer degraded. The high and low molecular weight polymer concentrations near the injection well were about 1499.50 mg/L and 0.50 mg/L, respectively, after injection of 0.64 PV polymer solution because the dynamic degradation time of the high molecular weight polymer was only 0.175 days, which is a very short time. Then, the high molecular weight polymer concentration near the injection well decreased to about 1218 mg/L, and the low molecular weight polymer concentration near the injection well increased to about 282 mg/L at 120 days after injection of 0.64 PV polymer solution. The average first-order degradation rate constant was about 0.0017 day^−1^, which is close to the first-order static degradation rate constant. This value agreed well with the fact that the polymer static degradation was dominant before the beginning of the subsequent water flooding in the polymer flooding experiment. It proved that the polymer static and dynamic degradation were described well in the designed simulator. Moreover, the total polymer concentration near the injection well remained at nearly 1500 mg/L during the 120 days. This demonstrated that the polymer degradation did not cause a change in the total polymer concentration, which is consistent with polymer theory. Additionally, the actual oil saturation distributions of each layer were examined by the saturation detector after the 0.64 PV polymer solution was injected in the experiment, as shown in Figure 14a. Figure 14b presents the oil saturation distribution obtained with the designed simulator. Some tiny differences were observed between the simulations, by comparing these two oil saturation distributions, which can mainly be attributed to the accuracy of the saturation detector, which was limited by laboratory conditions and influenced by the position and number of probes and experimental operations. Nevertheless, from the data obtained for the oil saturation distributions and the change trends in the oil saturation, the results can be also considered similar. Overall, the validation performed with polymer degradation including polymer static and dynamic degradations was positive and acceptable.

#### 3.3.2. Effect of Polymer Degradation on Production Indicators

**Figure 15:**
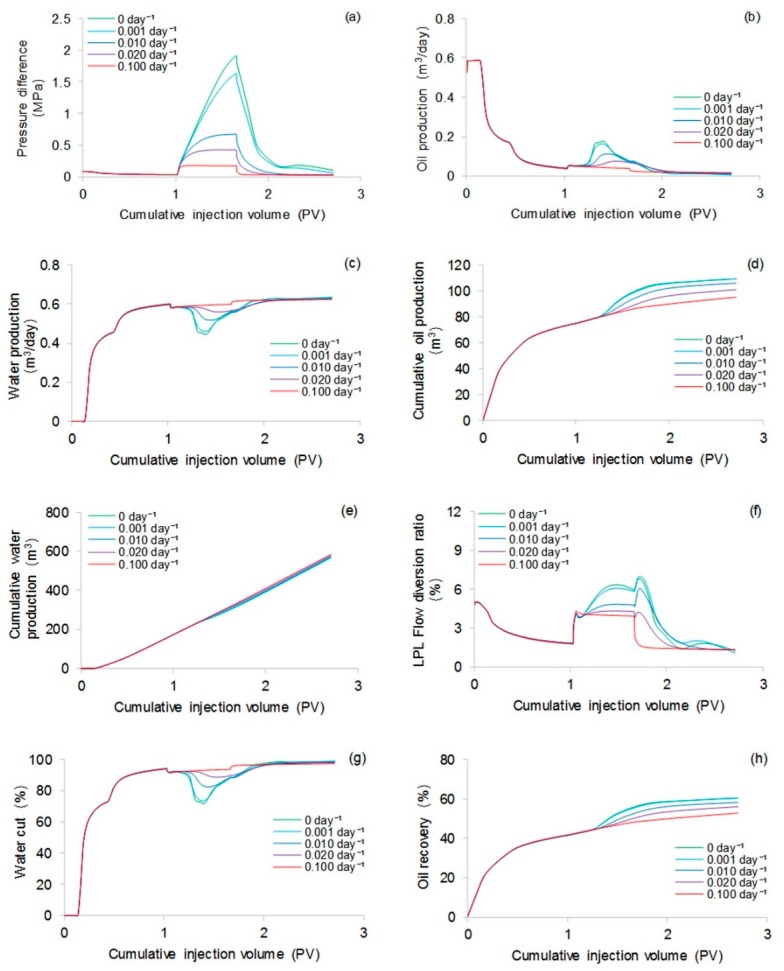
Comparative results for the production indicators including: (**a**) pressure difference, (**b**) oil production, (**c**) water production, (**d**) cumulative oil production, (**e**) cumulative water production, (**f**) LPL flow diversion ratio, (**g**) water cut, and (**h**) oil recovery with different first-order dynamic degradation rate constants.

**Figure 16:**
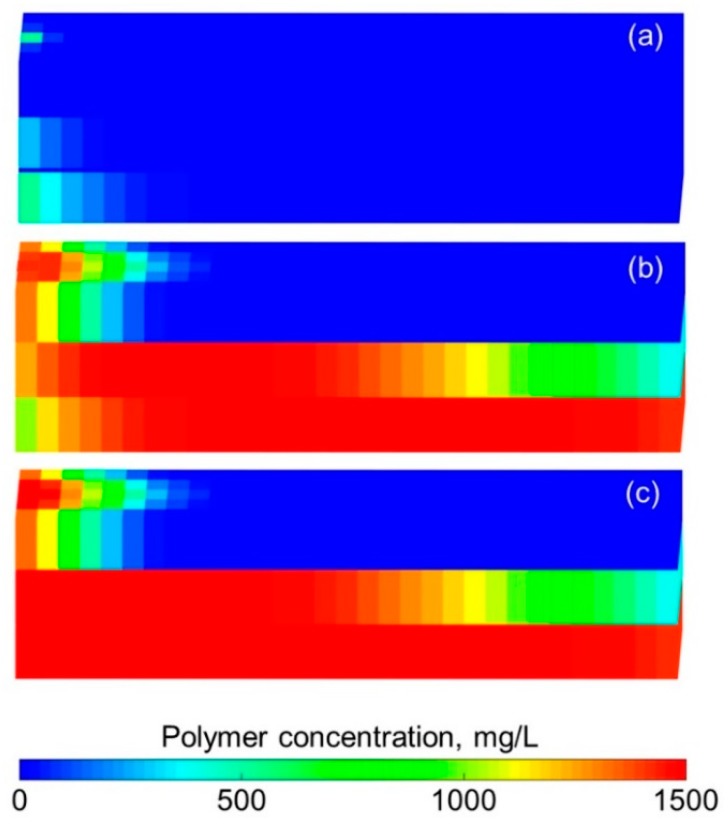
3D (**a**) high molecular weight, (**b**) low molecular weight, and (**c**) total polymer concentration distributions after cumulative injection volume of 1.67 PV of the simulation with a first-order dynamic degradation rate constant of 0.100 day^−1^.

**Figure 17:**
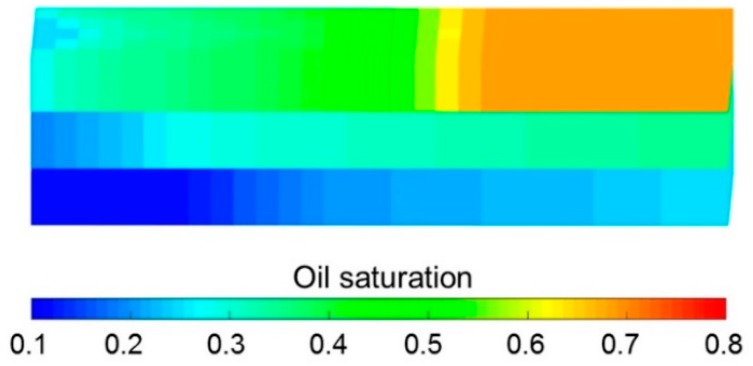
3D remaining oil saturation distribution after cumulative injection volume of 1.67 PV of the simulation with a first-order dynamic degradation rate constant of 0.100 day^−1^.

To analyze the effect of polymer degradation on production indicators, four more simulations were completed considering the first-order dynamic degradation rate constants of 0.001, 0.002, 0.010, and 0.100 day^−1^. The rest of the parameters were the same as those used in the validation case without polymer degradation. A similar method can be used to investigate the effect of polymer static degradation or both polymer static and dynamic degradations on production indicators, not conducted here because the polymer dynamic degradation occupied a dominant position or was even the only type of polymer degradation in typical polymer flooding. Figure 15 provides the comparative results of the production indicators at different first-order dynamic degradation rate constants. This figure indicates that the higher first-order dynamic degradation rate constant can more significantly impact on the production indicators. The reduction in production indicators caused by different first-order dynamic degradation rate constants, after a cumulative injection volume of 1.67 PV, is shown in Table 7. Notably, the oil recovery, one of the most critical production indicators, decreased 7.7% after a cumulative injection volume of 1.67 PV. Figure 16 shows the 3D high molecular weight, low molecular weight, and total polymer concentration distributions after a cumulative injection volume of 1.67 PV of the simulation, considering a first-order dynamic degradation rate constant of 0.100 day^−1^. From Figure 16a,b, the severe polymer degradation is obvious. After comparing Figure 16c and Figure 10b, we observed that the polymer leading edge in the simulation, with a first-order dynamic degradation rate constant of 0.100 day^−1^, was faster than that in the simulation without polymer degradation. The reason for this result is that a reduction in the polymer solution viscosity caused by polymer degradation led to an increase in the water-oil mobility ratio [53], resulting in the faster polymer leading edge. Figure 17 shows the 3D remaining oil saturation distribution after a cumulative injection volume of 1.67 PV of the simulation, with a first-order dynamic degradation rate constant of 0.100 day^−1^. Compared with Figure 11b, the remaining oil saturation of the simulation, with a first-order dynamic degradation rate constant of 0.100 day^−1^, was more than that of the simulation without considering polymer degradation. This result was also due to the reduction in the polymer solution viscosity caused by the polymer degradation, which led to the increase in the water-oil mobility ratio, resulting in unsatisfactory polymer flooding efficiency and more oil remaining in the reservoir.

## 4. Conclusions

In this research, physical experiments and numerical simulations were completed with the primary goals of studying the effect of polymer degradation on polymer flooding in heterogeneous reservoirs and providing theoretical and technical guidance for its application in the development of these reservoirs. The physical experiment results showed that the viscosity of the polymer solution and polymer concentration had a good power law relationship with a high correlation coefficient square of 0.98. The viscosity of the polymer solution increased with increasing polymer concentration. However, the viscosity of the polymer solution decreased with increasing shear rate, demonstrating that the rheological property of the polymer solution presented shear thinning performance. Moreover, the viscosity of the polymer solution decreased with increasing time due to the polymer degradation. The plots of the polymer static and dynamic degradations showed excellent exponential relationships with high correlation coefficient squares of 0.93 and 0.94, and the first-order static and dynamic degradation rate constants were 0.0017 day^−1^ and 0.0022 day^−1^, respectively. The first-order dynamic degradation rate constant was greater than the first-order static degradation rate constant due to additional polymer chain ruptures, which were caused by the additional shear stress during the polymer solution flow through sands, resulting in supplemental polymer degradation. Furthermore, the new 3D two-phase polymer flooding simulator that evaluated the polymer static and dynamic degradation was designed and validated with high accuracy and reliability by comparing the obtained results with the simulation results run by the commercial software, as well as with the results obtained from the polymer flooding experiments. Additionally, the effect of polymer degradation on the production indicators were analyzed using the deigned simulator. According to the simulation results, the production indicators were significantly influenced by the polymer degradation. In the case that used a first-order dynamic degradation rate constant of 0.100 day^−1^, the oil recovery, after a cumulative injection volume of 1.67 PV, was 7.7% lower than in the case without considering polymer degradation. The larger amount of remaining oil in the reservoir and the faster polymer leading edge that occurred due to a reduction in the polymer solution viscosity, caused by the polymer degradation, led to an increase in the water-oil mobility ratio, resulting in unsatisfactory polymer flooding.

Some methods have been proposed to minimize the effect of the HPAM degradation on polymer flooding [9,54,55,56]. A reduction in HPAM degradation should be ensured to increase polymer flooding efficiency. In addition to the methods used for reducing degradation, some polymers with low and even negative degradation rate constants in the model, whose viscosity slowly decreases and even increases during degradation, have been also proposed for use in polymer flooding to increase oil recovery, such as Xanthan gum, Scleroglucan, Schizophyllan, and cellulose nanocrystals [57,58,59,60]. In the future, we will focus on experiments on these polymers and incorporate their features into our designed simulator.

## Figures and Tables

**Table 1:** Measured properties of oil samples.

Parameter		Value
**Single carbon number, wt%**	C_3_	0.03
	C_4_	0.08
	C_5_	0.16
	C_6_	0.75
	C_7_	1.76
	C_8_	2.16
	C_9_	3.59
	C_10_	4.46
	C_11_	5.13
	C_12_	5.76
	C_12+_	76.12
**Density (45 °C), Kg/m^3^**		880
**Viscosity (45 °C), mPa∙s**		8.9

**Table 2:** Ion component concentrations in brine.

Ion Components	Concentration, mg/L
Na^+^ and K^+^	85.8
Ca^2+^	24.1
Mg^2+^	10.9
HCO_3_^−^	122
CO_3_^2−^	30
SO_4_^2−^	62.4
Cl^−^	53.2
TDS	388.4

**Table 3:** Polymer properties.

Properties	Description/Value
Type	HPAM
Molecular weight	2.5 × 10^7^
Solid content, wt%	91.2
Hydrolysis degree, %	26
Filtration factor	1.2
Dissolution rate, hour	<2
Insoluble matter, wt%	0.1
Granularity ≥1.0 mm, %	4.8
Granularity ≤0.2 mm, %	2.6

**Table 4:** Core sample parameters.

Parameters	Core Name		
	High Permeability Layer (HPL)	Middle Permeability Layer (MPL)	Low Permeability Layer (LPL)
Length, cm	29.89	29.9	29.89
Width, cm	4.43	4.45	4.44
Height, cm	2.5	2.5	2.5
Porosity, %	31.5	26.8	26.1
Permeability, mD	1250	600	120

**Table 5:** The reservoir property, fluid property, initial conditions and production data of the case without polymer degradation.

Input Parameters	Value	Input Parameters	Value
Initial porosity in HPL, MPL and LPL, fraction	0.258, 0.254, 0.249	Water formation volume factor	1.016
Initial permeability in x direction in HPL, MPL and LPL, mD	1250, 600, 120	Polymer concentration, mg/L	1500
Initial permeability in y direction in HPL, MPL and LPL, mD	1250, 600, 120	Inaccessible pore volume factor in HPL, MPL and LPL, fraction	0.05, 0.06, 0.08
Initial permeability in z direction in HPL, MPL and LPL, mD	125, 60, 12	Maximum polymer absorption in HPL, MPL and LPL, K_g_/K_g rock_	1.0 × 10^−5^, 1.1 × 10^−5^, 1.4 × 10^−5^
Reservoir temperature, °C	45	Residual resistance factor in HPL, MPL and LPL	1.35, 1.40, 2.20
Rock density in HPL, MPL and LPL, Kg/m^3^	2580, 2600, 2620	Initial reservoir pressure, MPa	10
Rock compressibility in HPL, MPL and LPL, MPa^−1^	2.8 × 10^−3^, 2.76 × 10^−3^, 2.7 × 10^−3^	Initial water saturation in HPL, MPL and LPL, fraction	0.261, 0.268, 0.315
Stock tank oil density, Kg/m^3^	880	Initial oil saturation in HPL, MPL and LPL, fraction	0.739, 0.732, 0.685
Initial oil viscosity, mPa∙s	8.9	Bottom hole pressure of production well, MPa	10
Oil compressibility, MPa^−1^	1.2 × 10^−3^	Injection rate, m^3^/day	0.64
Oil formation volume factor	1.068	Injected water during water flooding, PV	1.03
Initial water density, Kg/m^3^	1000	Injected polymer solution during polymer flooding, PV	0.64
Water viscosity, mPa∙s	0.69	Injected water during subsequent water flooding after polymer flooding, PV	1.06
Water compressibility, MPa^−1^	4.26 × 10^−4^		

**Table 6:** The parameters of the experimental simulation, which were different from those for the validation case without polymer degradation.

Parameters of the Experimental Simulation	Value
Length of the block along x, cm	0.96
Length of the block along y, cm	0.89
Length of the block along z in each layer, cm	2.5, 0.2, 2.5, 0.2, 2.5
Injection rate, cm^3^/min	0.64
Injected polymer solution during polymer flooding, PV	0.64
First-order static degradation rate constant, day^−1^	0.0017
First-order dynamic degradation rate constant, day^−1^	0.0022
Interval between polymer flooding and subsequent water flooding, day	120
Injected water during subsequent water flooding after polymer flooding, PV	4.16

**Table 7:** Reduction in production indicators caused by different first-order dynamic degradation rate constants after cumulative injection volume of 1.67 PV.

Production Indicators	First-Order Dynamic Degradation Rate Constant, Day^−1^			
	0.001	0.01	0.02	0.1
Pressure difference, MPa	0.28	1.24	1.48	1.74
Oil production, m^3^/d	0.00	0.00	0.01	0.03
Water production, m^3^/d	0.00	0.00	−0.01	−0.04
Water cut, %	−0.45	0.07	−1.26	−5.53
Cumulative oil production, m^3^	0.67	5.72	10.65	14.10
Cumulative water production, m^3^	−0.84	−6.66	−12.10	−15.90
HPL flow diversion ratio, %	0.80	2.35	1.96	−0.36
MPL flow diversion ratio, %	−0.90	−3.45	−3.61	−1.62
LPL flow diversion ratio, %	0.10	1.10	1.66	1.98
Oil recovery, %	0.37	3.15	5.87	7.77
